# MicroRNA Profiles Distinguishing Metastatic from Non-Metastatic Salivary Mucoepidermoid Carcinoma

**DOI:** 10.3390/jcm14144957

**Published:** 2025-07-13

**Authors:** Maria Eduarda Salles Trevizani, Fabio Albuquerque Marchi, Daniela Bizinelli, Katia Klug Oliveira, Fernanda Viviane Mariano, Cibele Pidorodeski Nagano, Felipe D’Almeida Costa, Clóvis Antonio Lopes Pinto, Luiz Paulo Kowalski, Silvia Vanessa Lourenço, Cláudia Malheiros Coutinho-Camillo

**Affiliations:** 1 International Research Center, A.C.Camargo Cancer Center, São Paulo 01508-010, Brazil; meduardatrevizani@gmail.com (M.E.S.T.); katiaklug@gmail.com (K.K.O.); 2 Department of Head and Neck Surgery, University of São Paulo Medical School, São Paulo 01246-903, Brazil; fabio.marchi@fm.usp.br; 3 Center for Translational Research in Oncology, Cancer Institute of the State of São Paulo (ICESP), São Paulo 01246-000, Brazil; 4 Interunit Graduate Program in Bioinformatics, Institute of Chemistry, University of São Paulo (USP), São Paulo 05508-900, Brazil; daniela.bizinelli@usp.br; 5 Department of Pathology, Faculty of Medical Sciences, University of Campinas (UNICAMP), São Paulo 13083-887, Brazil; fevimariano@gmail.com; 6 Department of Periodontology, Dental School, University Paris Cité, 75006 Paris, France; cibele.nagano@gmail.com; 7 Oral Medicine Department, Pitié-Salpêtrière Hospital, 75013 Paris, France; 8 Department of Anatomic Pathology, A.C.Camargo Cancer Center, São Paulo 01508-010, Brazil; felipe.costa@accamargo.org.br (F.D.C.); clovis.pinto@accamargo.org.br (C.A.L.P.); 9 Department of Head and Neck Surgery and Otorhinolaryngology, A.C.Camargo Cancer Center, São Paulo 01508-010, Brazil; lpkowalski@accamargo.org.br; 10 Department of General Pathology, Dental School, University of São Paulo, São Paulo 05508-000, Brazil; silvialourenco@usp.br; 11 National Institute of Science and Technology in Oncogenomics and Therapeutic Innovation, São Paulo 01508-010, Brazil

**Keywords:** mucoepidermoid carcinoma, microRNA profile, distant metastasis, lymph node metastasis

## Abstract

**Background/Objectives:** Mucoepidermoid carcinoma (MEC) is the most common malignant tumor of the salivary glands. Metastatic spread occurs in up to 80% of high-grade tumors; however, the mechanisms underlying this process are largely unknown. Large-scale microRNA (miRNA) expression profiling studies of human cancers have demonstrated that dysregulation of miRNA is frequently associated with many cancer types. This study aimed to investigate the miRNA profiles of metastatic and non-metastatic MECs. **Methods:** Using real-time RT-PCR (qPCR), we analyzed the expression of 377 miRNAs in four non-metastatic MECs, three MECs with lymph node metastasis, three MECs with distant metastasis, and two non-neoplastic human salivary glands. To identify differentially expressed miRNAs, bioinformatics analysis was performed using hierarchical clustering analysis. **Results:** The miRNA profile discriminated between non-neoplastic and tumor samples and between metastatic and non-metastatic tumors. Twelve miRNAs were differentially expressed between non-neoplastic and non-metastatic MECs. MEC analysis of non-neoplastic and lymph node metastases demonstrated that 10 miRNAs were differentially expressed. In non-neoplastic versus distant metastatic MECs, three miRNAs were differentially expressed: one downregulated and two upregulated. By comparing non-metastatic MECs with lymph node metastatic MECs, we identified 17 upregulated miRNAs. Considering non-metastatic MECs versus distant metastatic MECs, two miRNAs were upregulated. One miRNA was differentially expressed between lymph node metastatic and distant metastatic MECs. **Conclusions:** Our findings indicated that miRNA profiles may serve as valuable biomarkers for distinguishing the metastatic potential of salivary MECs, warranting further investigation to validate their utility in clinical practice.

## 1. Introduction

Mucoepidermoid carcinoma (MEC) is the most common malignant tumor of the salivary glands and especially affects the parotids. It comprises 10–15% of salivary gland tumors (SGTs) and 30% of malignant SGTs [1,2,3]. MEC is derived from the main duct segment and composed of mucous, intermediate, and epidermoid cells in varying combinations [1]. Histological grading, which can be categorized as low, intermediate, or high, is widely recognized for its prognostic significance. This grading is based on several factors, including architectural formation, cytological characteristics, evidence of perineural invasion, and presence of necrosis [1,4,5].

Lymph node metastasis is frequently observed in high-grade MECs, occurring in up to 80% of the cases. Additionally, the presence of distant metastasis has been reported in up to 63% of these tumors, serving as a significant predictor of poor prognosis [6,7]. However, the mechanisms underlying the metastatic process remain largely unknown, and metastasis can develop several years after initial diagnosis [4,8,9,10].

microRNAs (miRNAs) are approximately 22-nucleotide non-coding RNA molecules that post-transcriptionally regulate gene expression and are involved in various biological processes [11,12]. Large-scale miRNA expression profiling studies in human cancers have revealed that dysregulation of miRNAs is frequently associated with various cancer types. However, few studies have investigated the role of miRNAs in the development and progression of SGTs (for review, see Han et al., 2014; dos Santos et al., 2021; El-Husseiny et al. 2023) [13,14,15].

This study aimed to identify a miRNA profile that distinguishes metastatic from non-metastatic MECs. This profiling could not only enhance our understanding of the molecular basis of these lesions but also improve patient management based on the aggressiveness of the disease, allowing the stratification of the patients’ risk, increasing the accuracy of pre-surgical diagnosis and aiding in the planning of the appropriate treatment.

## 2. Materials and Methods

### 2.1. Tissue Samples

Paraffin-embedded tissue samples were obtained from 10 MECs (4 non-metastatic, 3 with lymph node metastasis, and 3 with distant metastasis) and from 2 non-neoplastic salivary gland samples. All samples were sourced from the Department of Pathology at A.C.Camargo Cancer Center in São Paulo, Brazil. All retrieved cases were of patients who had been under treatment at the hospital for a minimum of 5 years or until death associated with cancer progression. We included patients who had undergone primary surgical excision without prior radiotherapy or chemotherapy. Demographic, clinical, and histological details of the patients are presented in Table 1. This study was approved by the Institutional Ethics Committee on 23 April 2019 (protocol number 2700/19).

### 2.2. RNA Isolation

Total RNA was obtained by scraping MEC and non-neoplastic tissues from 3 µm paraffin-embedded sections (10 sections from each sample) and extracted using a RecoverAll kit (Ambion, Austin, TX, USA), according to the manufacturer’s instructions, and quantified using a Nanodrop 1000 (Nanodrop, Waltham, MA, USA).

### 2.3. Real Time RT-PCR (qPCR): miRNA Expression

Total RNA (300 ng) was reverse-transcribed from neoplastic and non-neoplastic samples using the TaqMan microRNA Reverse Transcription kit and the Megaplex Reverse Transcription primer pool A (Applied Biosystems, Foster City, CA, USA).

The RT product was pre-amplified using Megaplex PreAmp Primers pool A and TaqMan PreAmp Master Mix according to the manufacturer’s instructions (Applied Biosystems, Foster City, CA, USA). PCR amplification was performed with an ABI 7900HT Sequence Detection System (Applied Biosystems, Foster City, CA, USA) using TaqMan Universal Master Mix (Applied Biosystems, Foster City, CA, USA) under default thermal cycling conditions.

miRNA expression was evaluated using the TaqMan Array Human MicroRNA A Card, containing 377 miRNAs and 7 controls (Applied Biosystems, Foster City, CA, USA). The relative miRNA expression level was normalized based on the expression of the reference miRNA (miR-27b). The reference miRNA was selected using NormFinder software version 21 (https://www.moma.dk/software/normfinder (access date: 8 June 2025), Department of Molecular Medicine (MOMA), Aarhus N, Denmark). The relative miRNA expression level was also normalized based on the expression of a calibrator sample (a pool of two non-neoplastic salivary gland samples). The final results, expressed as *n*-fold differences in miRNA expression relative to the expression of reference miRNAs and the calibrator sample, were determined in exponents as follows [16].

Relative expression (Rq) = 2 ^−(ΔCt sample − ΔCt calibrator)^ where ΔCt values of the sample and calibrator are determined by subtracting the average Ct value of the target miRNAs from the average Ct value of the reference miRNA. Ct values above 37 were excluded from the downstream analyses. To enable group comparisons and consistent interpretation of expression differences, Rq values were subsequently transformed using the formula log_2_FC = log_2_(Rq), and results were expressed as log_2_ fold changes (log_2_FC).

### 2.4. Statistical Analysis

log_2_-transformed miRNA expression data was used to quantify the differential expression as fold change, with thresholds set at ≥2 for upregulation and ≤−2 for downregulation. Subsequent analyses were conducted using RStudio software (version 2024.12.1) with R (version 4.4.0; https://www.r-project.org, accessed on 10 July 2024). Group comparisons were assessed using Student’s *t*-test, with statistical significance defined as *p* < 0.05.

The data were visualized using the ggplot2 package version 3.5.2 (https://www.tidyverse.org (accessed on 10 July 2024)). Unsupervised hierarchical clustering was conducted using the ComplexHeatmap package [17], available through Bioconductor (www.bioconductor.org (accessed on 10 July 2024)), to explore expression patterns across sample groups.

## 3. Results

**Figure 1:**
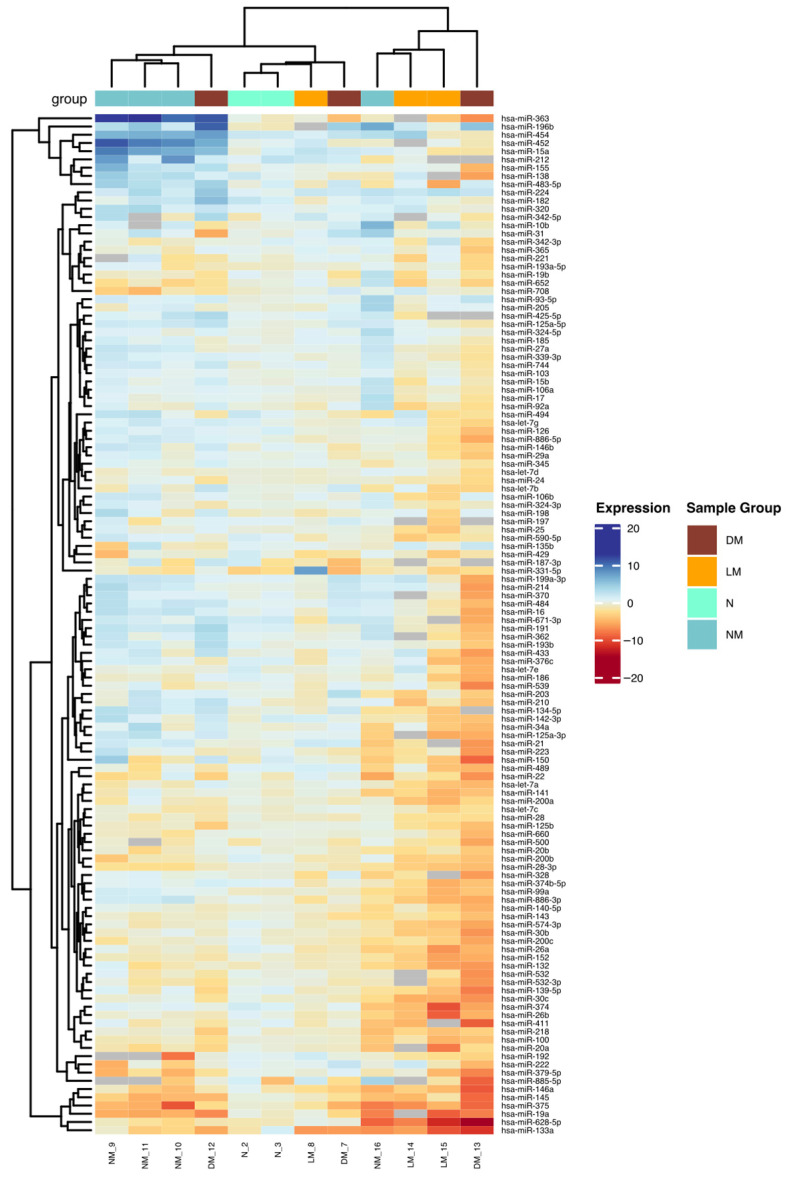
Hierarchical clustering analysis of miRNA expression in metastatic and non-metastatic mucoepidermoid carcinoma (MEC) and non-neoplastic salivary gland samples. The data sets were normalized based on the expression of a reference miRNA (hsa-miR-27b) and a pool of two non-neoplastic salivary gland samples. The relative upregulation and downregulation of miRNAs are indicated by red and blue, respectively. DM, MEC with distant metastasis; LM, MEC with lymph node metastasis; N, normal salivary gland; NM, non-metastatic MEC.

Of the 377 miRNAs included in the array panel, 119 were not detected in salivary gland tissue samples. The expression profiles of the remaining miRNAs effectively distinguished between non-neoplastic tissue, non-metastatic MECs, MECS with lymph node metastasis, and distant metastasis, as illustrated in Figure 1 and detailed in Table 2.

When comparing non-neoplastic salivary glands to non-metastatic MECs, 12 miRNAs were differentially expressed, with 7 downregulated (miR-125a-5p, miR-27a, miR-191, miR-199a-3p, miR-103, miR-196b, and miR-454) and 5 upregulated (miR-28-3p, miR-145, miR-19a, miR-186, and miR-375) miRNAs in non-neoplastic samples.

A comparison between non-neoplastic salivary glands and lymph node metastatic MECs revealed 10 dysregulated miRNAs, including 8 upregulated (miR-133a, miR-886-3p, miR-590-5p, miR-200b, miR-671-3p, miR-191, miR-328, and miR-339-3p) and 2 downregulated (miR-224 and miR-324-5p) miRNAs. In distant metastatic MECs compared to non-neoplastic glands, three miRNAs were dysregulated: miR-19b and miR-494 were upregulated, and miR-134-5p was downregulated.

A comparison between non-metastatic MECs and those with lymph node metastasis revealed 17 upregulated miRNAs in the non-metastatic group. Among these, highly upregulated miRNAs (fold change ≥ 8) included miR-886-3p, miR-590-5p, miR-210, miR-374b-5p, and miR-191. Moderately upregulated miRNAs (fold change between 4 and 7.9) included miR-27a, miR-16, miR-339-3p, miR-671-3p, miR-484, miR-324-3p, miR-744, miR-886-5p, miR-106a, and miR-106b. Finally, mild upregulation (fold change between 2 and 3.9) was observed for miR-125a-5p and miR-24.

Compared to distant metastatic MECs, non-metastatic tumors exhibited upregulation of miR-27a and miR-24. In addition, miR-134-5p was the only miRNA that was differentially expressed when contrasting lymph nodes and distant metastases.

## 4. Discussion

The mechanisms underlying the metastasis of SGTs remain largely unknown, partly because of their rarity. Additionally, metastasis can occur years after diagnosis [4,8,9,10]. Adenoid cystic carcinoma, salivary duct carcinoma, and MEC are associated with a high risk of metastasis, and distant metastasis is frequently observed in the lungs, bones, brain, liver, and skin [8,10].

Several studies have addressed the clinicopathological predictive factors for distant metastasis in SGTs, including tumor size, histological grade, and perineural invasion [9,18,19,20]. However, the molecular mechanisms underlying the onset of salivary gland metastasis remain unclear.

Various molecular techniques have been used to evaluate SGTs, including immunohistochemistry, fluorescent/chromogenic in situ hybridization, microarray analyses, sequencing, and reverse transcription-polymerase chain reaction (RT-PCR) [21,22]. Some studies have evaluated the role of miRNAs in the development and progression of SGTs [23,24,25,26,27,28,29,30,31,32].

miRNAs are stably expressed in different types of body fluids (serum, plasma, saliva, and urine) and are less susceptible to degradation, representing promising diagnostic and prognostic biomarkers for cancer [33,34]. Large-scale miRNA expression profiling studies of human cancers have demonstrated that dysregulation of miRNAs is frequently associated with many types of cancer, such as breast cancer and colorectal cancer [13,35].

In the present study, we analyzed the miRNA profiles of non-metastatic MEC, MEC with lymph node metastasis, and MEC with distant metastasis in comparison with non-neoplastic salivary gland samples and identified 34 differentially expressed miRNAs. Binmadi et al. (2018) [36] evaluated six MEC samples in comparison with non-neoplastic samples using the same methodology, TaqMan Array (TLDA). They observed the upregulation and downregulation of several miRNAs in MEC samples, and some miRNAs demonstrated concordant results with those obtained in our study: miR-19a and miR-375 were downregulated and miR-27a, miR-199a-3p, miR-103, and miR-196b were upregulated in MEC when compared with those in normal tissue samples. Naakka et al. (2022) [37] also observed miR-375 downregulation in MEC using microarray technology.

Matse et al. (2013) [27] applied the TaqMan Array methodology to saliva from patients with parotid tumors and identified 57 miRNAs that were differentially expressed between malignant and benign samples. Most of these miRNAs were upregulated in malignant samples compared with those in benign samples, thus emphasizing the potential of miRNAs as key biomarkers in determining tumor prognosis. Notably, miR-324-5p, miR-744, miR-16, miR-145, miR-324-3p, and miR-200b emerged as examples of miRNAs that were highly expressed in malignant samples. Furthermore, the deregulation of these miRNAs was identified in the MEC samples examined in the present study.

Denaro et al. (2019) [38] used NanoString technology to evaluate the miRNA profile in 10 benign versus 14 malignant SGTs (including 6 MECs) and observed 46 differentially expressed miRNAs. miR-106a and miR-106b, constituents of the miR-17-92 cluster, were upregulated in both Denaro’s study and the current study. The observed dysregulation of these miRNAs in SGTs suggests their potential involvement not only in tumor pathogenesis but also in regulating other cancer-related processes. This aligns with the findings reported by Mitani et al. (2013) [25], in which the upregulation of the miR-17-92 cluster was associated with the aggressive behavior of ACC tumors.

Using microarray analysis, Lu et al. (2019) [39] identified a total of 3612 mRNAs, 3091 lncRNAs, and 284 circRNAs altered during the pathogenesis of MEC, which has succeeded in increasing the knowledge of the molecular mechanisms of MEC, and the expression of these molecules may be possible targets for intervention therapy. One mechanism involves the distinctive expression of circRNAs, which influences gene expression and pathways associated with metabolism. In this study, circ012342 was identified as potentially targeting specific miRNAs, including miR-107 and miR-214-3p, that are involved in oncogenic processes. This exploration sheds light on potential molecular mechanisms underlying these interactions.

Other authors studying salivary adenoid cystic carcinoma (SACC) have evaluated miRNA profiles using different methodological approaches [30,31,40,41,42]. Andreasen et al. (2018) [30] and Han et al. (2018) [31] demonstrated the relationship of miRNAs in cancer-related biological processes, which implicate differences in the overall and recurrence-free survival rates of patients. Han et al. (2018) [31] performed a combined miRNA-mRNA regulatory network analysis to determine the genes with carcinogenic potential in SACC and identified molecules that were associated with oncogenic processes.

Considering the differentially expressed miRNAs, according to the literature, their dysregulation might be related to oncogenic processes in different tumors. Han et al. (2014) [13] conducted a meta-analysis to evaluate the diagnostic value of miRNAs in detecting cancer metastasis and observed that miR-145 overexpression was involved in suppressing tumor formation and expression of cancer stem cell markers, thereby inhibiting metastasis. We also observed that miR-145 was upregulated in non-neoplastic samples compared with that in non-metastatic samples, confirming that its inadequate expression may facilitate oncogenic processes. Naakka et al. (2022) [37] also observed miR-145 downregulation in MECs. Abdolrahmani et al. (2022) [43] reported lower expression of miR-145 in MEC than in normal salivary gland tissue and confirmed a negative correlation between miR-145 expression level and MUC1 expression and histologic grade.

Consistent with our results, some studies have demonstrated the association of miRNAs with the suppression of migration and invasion of different tumors, such as miR-24, miR-16, and miR-210, which are overexpressed in non-metastatic samples compared with those in metastatic samples in nasopharyngeal carcinoma, breast cancer, and gastric cancer, respectively [44,45,46]. Regarding miRNAs that enhance proliferation, migration, and invasion, overexpression of mir-19a, miR-125a-5p, miR-484, and miR-744 has also been observed in metastatic samples from colorectal cancer, head and neck cancer, prostate cancer, and lung cancer, respectively, suggesting a relationship between these miRNAs and metastasis [47,48,49,50].

It is important to emphasize that few studies have addressed the expression of miRNAs implicated in the metastasis of SGTs. These studies were predominantly conducted in adenoid cystic carcinomas. In addition, the reported studies employed various methodological approaches and sample sources, which may have resulted in a heterogeneous array of observed miRNAs. Although qRT-PCR is regarded as the gold standard for quantifying miRNA expression, owing to its rapidity, specificity, sensitivity, and cost-effectiveness, it has some limitations, including primer design challenges and requirements for effective endogenous normalization.

Our findings reveal a set of miRNAs that are differentially expressed in MEC samples, suggesting that miRNA profiles could potentially discriminate the metastatic potential of these tumors. At first moment, the miRNA profile could play a role in the risk stratification of the patients and, in conjunction with imaging and histological tests, increase the accuracy of pre-surgical diagnosis and aiding in the planning of the appropriate treatment. At a second stage, the restoration of miRNA expression might represent an approach for targeted therapy to prevent tumor progression, in addition to the regular treatment. However, we acknowledge the limitations inherent to our study, including the small sample size and its retrospective nature, which underscore the need for further research to substantiate the clinical utility of these biomarkers.

## Figures and Tables

**Table 1:** Summary of demographic, clinical, and pathological characteristics of the patients with mucoepidermoid carcinoma (MEC).

ID	Age (Years)	Sex	Race	Tumor	Histological Grade	Tumor Site	Vascular Invasion	Perineural Invasion	Lymph Node
MEC9	49	Male	White	Non-metastatic MEC	Low	Parotid	No	No	No
MEC10	15	Female	Non-white	Non-metastatic MEC	Intermediate	Parotid	No	Yes	No
MEC11	11	Female	Non-white	Non-metastatic MEC	Low	Parotid	-	-	No
MEC16	72	Male	White	Non-metastatic MEC	High	Parotid	No	No	No
MEC8	53	Male	White	Lymph node metastatic MEC	High	Parotid	No	No	Yes
MEC14	12	Male	Non-white	Lymph node metastatic MEC	-	Parotid	-	-	Yes
MEC15	68	Male	-	Lymph node metastatic MEC	High	Parotid	No	No	Yes
MEC7	30	Female	White	Distant metastatic MEC	Intermediate	Parotid	Yes	Yes	Yes
MEC12	-	Male	-	Distant metastatic MEC	-	Parotid	-	-	-
MEC13	60	Male	White	Distant metastatic MEC	High	Parotid	No	Yes	Yes

**Table 2:** Differentially expressed miRNAs comparing metastatic mucoepidermoid carcinoma (MEC), non-metastatic MEC, and non-neoplastic salivary gland tissue.

**Non-Neoplastic × Non-Metastatic MEC**		
microRNA	Fold Change	*p*-Value
hsa-miR-28-3p	3.97	0.000
hsa-miR-145	16.83	0.003
hsa-miR-19a	39.24	0.003
hsa-miR-186	3.11	0.010
hsa-miR-375	67.85	0.021
hsa-miR-125a-5p	−2.91	0.001
hsa-miR-27a	−3.59	0.002
hsa-miR-191	−2.12	0.018
hsa-miR-199a-3p	−2.47	0.028
hsa-miR-103	−1.56	0.035
hsa-miR-196b	−31.70	0.038
hsa-miR-454	−11.35	0.040
**Non-neoplastic × lymph node metastatic MEC**		
microRNA	Fold change	*p*-value
hsa-miR-328	3.41	0.017
hsa-miR-191	3.78	0.017
hsa-miR-133a	118.23	0.030
hsa-miR-200b	9.21	0.032
hsa-miR-590-5p	10.31	0.032
hsa-miR-886-3p	13.62	0.040
hsa-miR-339-3p	2.13	0.041
hsa-miR-671-3p	6.24	0.043
hsa-miR-324-5p	−1.53	0.021
hsa-miR-224	−3.54	0.030
**Non-neoplastic × distant metastatic MEC**		
microRNA	Fold change	*p*-value
hsa-miR-19b	4.57	0.004
hsa-miR-494	6.96	0.021
hsa-miR-134-5p	−4.55	0.024
**Non-metastatic MEC × lymph node metastatic MEC**		
microRNA	Fold change	*p*-value
hsa-miR-191	7.99	0.000
hsa-miR-125a-5p	3.23	0.002
hsa-miR-27a	4.55	0.002
hsa-miR-16	6.77	0.004
hsa-miR-339-3p	3.84	0.005
hsa-miR-590-5p	8.53	0.010
hsa-miR-671-3p	5.41	0.018
hsa-miR-886-3p	20.25	0.019
hsa-miR-484	4.64	0.022
hsa-miR-24	2.06	0.025
hsa-miR-374b-5p	8.88	0.026
hsa-miR-324-3p	3.43	0.030
hsa-miR-210	8.21	0.032
hsa-miR-744	2.38	0.032
hsa-miR-886-5p	4.58	0.033
hsa-miR-106a	2.97	0.037
hsa-miR-106b	6.87	0.047
**Non-metastatic × distant metastatic MEC**		
microRNA	Fold change	*p*-value
hsa-miR-27a	6.00	0.004
hsa-miR-24	3.13	0.048
**Lymph node metastatic MEC × distant metastatic MEC**		
microRNA	Fold change	*p*-value
hsa-miR-134-5p	−18.52	0.036

## Data Availability

The authors confirm that the data supporting the findings of this study are available within the article.

## Abbreviations

MEC	mucoepidermoid carcinoma
miRNA	microRNA
RT-PCR	reverse transcription–polymerase chain reaction
SGT	salivary gland tumor
